# Measurement of craniocaudal catheter displacement between fractions in computed tomography–based high dose rate brachytherapy of prostate cancer

**DOI:** 10.1120/jacmp.v8i4.2415

**Published:** 2007-09-17

**Authors:** Yongbok Kim, I‐Chow J. Hsu, Jean Pouliot

**Affiliations:** ^1^ Department of Radiation Oncology University of California–San Francisco, Comprehensive Cancer Center 1600 Divisadero Street, Suite H1031 San Francisco CA 94143‐1708

**Keywords:** catheter displacement, HDR brachytherapy, prostate cancer, bony marker, center of two gold markers

## Abstract

The objective of the present work was to measure the craniocaudal displacement of catheters occurring between consecutive fractions of transrectal ultrasound (TRUS)‐guided high dose rate (HDR) prostate brachytherapy. Ten consecutive patients were treated with 2 fractions of 9.5‐Gy TRUS‐guided HDR brachytherapy, with dental putty being used for the fixation of catheters. For each patient, a computed tomography (CT) scan with 3‐mm slice thickness was acquired before each of the 2 fractions. Two different references were used to measure the catheter displacement between fractions: the ischial bone as a bony marker (BM) and the center of two gold markers (COGM) implanted in the prostate. Catheter displacement was calculated by multiplying the thickness of the CT slice by the difference in number of CT slices between the reference slice and the slice containing the tip of a catheter. The average magnitude of caudal catheter displacement was 2.7 mm (range: −6.0 mm to 13.5 mm) for the BM method and 5.4 mm (range: −3.75 mm to 18.0 mm) for the COGM method. The measurement data obtained from the BM and COGM methods verified that prostate movement and catheter displacement both occurred independently between fractions. The most anterior and medial two catheters (catheter positions 8 and 12) had the greatest tendency to be displaced in the caudal direction because they were located at the most distant position from the fulcrum, making them susceptible to rotation of the dental putty in the lateral plane because of the movement of the patients’ legs between fractions. In conclusion, the combination of the BM and COGM methods can demonstrate prostate and catheter movement relative to the BM between fractions. Our technique found a pattern of catheter displacement. Based on that finding, further improvement of our results may be possible by modification of our current technique.

PACS number: 87.53.Jw

## I. INTRODUCTION

High dose rate (HDR) brachytherapy can deliver a very conformal radiation dose to the prostate with catheters inserted into the tumor. Recently, computed tomography (CT) and magnetic resonance imaging (MRI) were introduced into HDR brachytherapy planning. The anatomic information is displayed together with the dose distribution within the target and the organs at risk (OARs). Three‐dimensional (3D) treatment planning significantly improves display of dosimetric information and allows for dwell times to be adjusted to improve coverage of the target while sparing critical organs adjacent to the target.^(1)^

Another advancement made in HDR brachytherapy is the development of the inverse planning software that allows dwell time distribution to be optimized, providing the desired dose distribution based on the prescribed dose constraints.^(^ ^2^ ^–^ ^4^ ^)^ Furthermore, functional imaging information from magnetic resonance spectroscopy can be used for treatment planning to better identify dominant intraprostatic malignant lesions.^(5)^

Despite these advantages, dose uncertainties in HDR brachytherapy for prostate cancer remain. We recently addressed the dosimetric impact of prostate volume change resulting from the trauma caused by the insertion of catheters and from resolution of edema between fractions,^(6)^ and the dose uncertainty attributable to the intrinsic characteristics of the finite CT slice thickness.^(7)^ These factors translate into a discrepancy between the source dwell positions observable on planning CT or MRI images and the actual dwell positions during dose delivery by the afterloader.

In the present study, we measured the craniocaudal catheter displacement attributable to patient and prostate motion between fractions by acquiring CT scans before each fraction for 10 patients.

## II. MATERIALS AND METHODS

For this study, we recruited 10 consecutive patients (identified here as patients A – J). Each patient was implanted with 16 catheters for except for patient A (14 catheters) and patient I (18 catheters). The total number of catheters depended on prostate size and need to cover the entire tumor volume. The size of the prostate was determined from the planning target volume in the CT‐based HDR planning procedure. The mean values ± standard deviation for the volume of prostate implanted with 16 catheters was $37.7\pm 6.2\;{\text{cm}}^{3}$. The prostate volume for patient A was 23.1 cm^3^, and for patient I, it was 136.1 cm^3^.

### A. Treatment procedures

**Figure 1:**
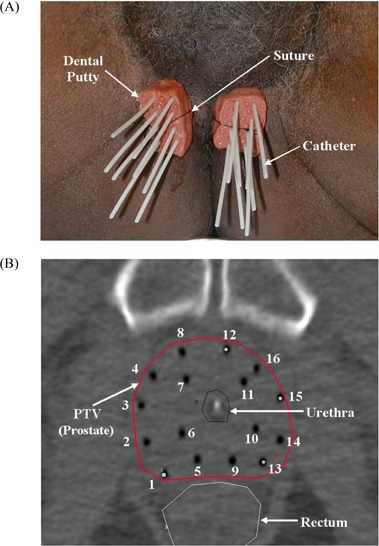
(A) Fixation of 16 catheters on the perineum using dental putty. (B) Typical pattern of 16 catheters inserted into the prostate (axial computed tomography slice for mid‐gland of prostate).

At our institute, HDR prostate brachytherapy boost is performed in two 9.5‐Gy fractions after 45 Gy of external‐beam radiotherapy. During the procedure, a physician inserts Flexi‐guide catheters (Best Medical, Springfield, VA) into the prostate using a freehand transrectal ultrasound (TRUS)–guided technique. Instead of conventional prefabricated template, an in‐house customized catheter fixation technique using dental putty [Fig. 1(A)]^(8)^ was developed and is used for this procedure.

First, the entry point of each catheter is marked on the perineum so as to avoid hitting the ischium and the urethra. The entry points are separated into two groups on either side of the median raphe. At the marked entry points, the catheters are inserted under TRUS guidance in a “freehand” manner without a template.

Four inner catheters are placed around the urethra: the two most anteromedial catheters first, followed by two catheters placed just posterior to the first two catheters. The remaining outer catheters are evenly distributed on the periphery of the prostate.

Once all catheters are inserted, the obturators are removed, and the Luer locks on the Flexi‐guides are trimmed off. The friction collars are placed on each catheter. Dental molding putty (Reprosil Type I, very high viscosity: L.D. Caulk Division, Dentsply International, York, PA) is placed around the catheters and friction collars for each group. This particular putty hardens in 3 minutes after its two components are mixed. Finally, the whole system for each group of catheters is secured against the perineum by a suture [Fig. 1(A)].

As compared with the conventional prefabricated template method, this freehand TRUS‐guided technique provides more freedom for catheter distribution to be adapted to the patient's anatomy. Hence, the catheters can be closer together at some levels and farther apart at other levels to increase dose conformality to target while sparing normal structures.

The entire treatment procedure—consisting of catheter insertion, CT‐based treatment planning (Plato, version 14.2: Nucletron, Veenendaal, Netherlands), and treatment delivery—is performed over 24 hours. The first fraction is delivered in the early afternoon on the first day, and the second fraction is delivered in the morning of the second day.^(^ ^6^ ^,^ ^8^ ^)^ A single treatment plan is used for both fractions. Treatment plans are obtained using our in‐house anatomy‐based inverse planning algorithm (Inverse Planning Based on Simulated Annealing), which optimizes the dwell times once the dose constraints and the prescription are specified.^(^ ^2^ ^–^ ^4^ ^)^

### B. General catheter pattern

As seen in Fig. 1(B), the 16 catheters are generally inserted into the prostate in 4 rows by 4 columns. Labeling proceeds from right first to left fourth column. The numbering scheme for the catheters is also shown in Fig. 1(B). The catheters fixed by the dental putty are not parallel as in the conventional prefabricated template technique. They sometimes converge or diverge to cover the entire target volume, based on the TRUS image. In Fig. 1(B), the midline of the prostate was satisfactorily covered by the catheters on the second (positions 5 – 8) and third columns (positions 9 – 12), even though no catheters are located at the midline of perineum [Fig. 1(A)].

### C. Measurement of catheter displacement

To measure the craniocaudal catheter displacement between fractions, a second CT scan with a 3‐mm slice spacing and thickness (Somatom Emotion: Siemens Medical Solutions, Malvern, PA) was obtained for each patient with implanted catheters on the morning of the second day before delivery of the second treatment. The spiral CT modality with 3‐mm collimation and reconstruction thickness, 6 mm‐per‐rotation bed speed, and 1‐s gantry rotation period was used for the pelvic scan. The CT gantry angle was 0 degrees, the field of view was 15×15 cm, and the image resolution was 1.24×1.24. Patient setup for the pelvic CT scan was head first in the supine position with a pillow under the knees, and hands on chest.

All catheter displacement measurements on the first‐ and second‐day axial CT images were made by one observer. The tips of the catheters were identified by locating the end of the air column in each catheter on the CT image.

The air column in a catheter appears as a black dot on axial CT image. Sometimes, the size of the black dot on the last axial CT image containing the tip of a catheter is smaller than expected because the reconstruction volume for that CT slice does not fully contain the tip of the catheter. Hence, the tip of the catheter is assumed to be located between the current slice and the previous one.^(7)^ For instance, if the size of black dot shown on CT slice (*i*) that contains the tip of a catheter is not as large as expected, the tip of the catheter is considered to be located on a CT slice assigned a fractional number, for example (*i*) – 0.5. The catheter depth calculated on the (*i*)th CT slice is therefore reduced by 1.5 mm to account for the assigned fractional CT slice number.

The measured catheter insertion depth is the distance from the tip of each catheter to a reference CT slice. Two different references were used for each measurement. In the first method, the most inferior CT slice containing the ischial bone [bony marker (BM)] was chosen as the reference CT slice. For the second method, the center of the two gold seed markers (COGM) implanted in the prostate (one at the base and the other at the apex of the gland) was used to determine the reference CT slice. The displacement was calculated by multiplying the difference between the catheter depths measured on the CT scans taken on days 1 and 2 by the 3‐mm CT slice thickness, as follows:

(1) $${\left(\text{Catheter}\;\text{depth}\right)}_{\text{Day}1}={\left[{\text{CT}\_\text{Slice}}_{\text{CathTip}}-({\text{CT}\_\text{Slice}}_{\text{GM}1}+{\text{CT}\_\text{Slice}}_{\text{GM}2})/2\right]}_{\text{Day}1}\times 3\;\text{mm}$$

(2) $${\left(\text{Catheter}\;\text{depth}\right)}_{\text{Day}2}={\left[{\text{CT}\_\text{Slice}}_{\text{CathTip}}-({\text{CT}\_\text{Slice}}_{\text{GM}1}+{\text{CT}\_\text{Slice}}_{\text{GM}2})/2\right]}_{\text{Day}2}\times 3\;\text{mm}$$

(3) $$\text{Catheter}\;\text{displacement}={\left(\text{Catheter}\;\text{depth}\right)}_{\text{Day}2}-{\left(\text{Catheter}\;\text{depth}\right)}_{\text{Day}1}$$

In the present study, a positive displacement means that the catheter has moved inferiorly from day 1 to day 2 (caudal displacement), and a negative displacement means that the catheter has moved deeper into the patient superiorly (cranial displacement). Descriptive statistics were used to analyze the measurements for each patient and each catheter position. Measurements of all 160 catheters were performed again by another observer to assess inter‐observer differences in the measured catheter displacements.

## III. RESULTS

**Figure 2:**
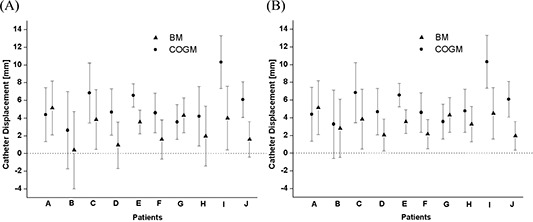
(A) Measurement of craniocaudal catheter displacement using the bony markers (BM) method and the center of two gold markers (COGM) method for 10 patients. In each patient's graph, the filled triangle and filled circle represent the mean values of the BM and COGM measurement displacements from day 1 to day 2 respectively, and the error bars show one standard deviation. (B) All data were changed into absolute values and re‐plotted.

Fig. 2(A) shows a graph of the measurement data (mean±standard deviation) by patient, and these data are summarized in Table 1 for all 10 patients. All measurement data were also converted into absolute values and re‐plotted as shown in Fig. 2(B).

**Table 1:** Statistics for craniocaudal catheter displacement measurements for 10 patients

						95% CI	
Patient	Mean	SD	Median	Minimum	Maximum	From	To
Measurements by the bony marker (BM) method							
A	5.1	3.0	6.0	1.5	12.0	3.4	6.9
B	0.4	4.4	−0.8	−6.0	13.5	−2.0	2.7
C	3.8	3.4	3.0	0.0	15.0	2.0	5.6
D	0.9	2.6	0.8	−1.5	6.0	−0.5	2.3
E	3.6	1.3	3.8	1.5	6.0	2.9	4.3
F	1.6	2.2	1.5	−3.0	6.0	0.4	2.8
G	4.3	2.0	3.0	1.5	9.0	3.3	5.4
H	2.0	3.4	3.0	−4.5	6.0	0.2	3.8
I	4.0	3.6	4.5	−4.5	12.0	2.2	5.8
J	1.6	2.0	1.5	−1.5	6.0	0.5	2.7
Measurements by the center of two gold markers (COGM) method							
A	4.4	3.0	5.3	0.8	11.3	2.6	6.1
B	2.6	4.4	1.5	−3.8	15.8	0.3	5.0
C	6.8	3.4	6.0	3.0	18.0	5.0	8.6
D	4.7	2.6	4.5	2.3	9.8	3.3	6.1
E	6.6	1.3	6.8	4.5	9.0	5.9	7.3
F	4.6	2.2	4.5	0.0	9.0	3.4	5.8
G	3.6	2.0	2.3	0.8	8.3	2.5	4.6
H	4.2	3.4	5.3	−2.3	8.3	2.4	6.0
I	10.3	3.0	10.5	6.0	18.0	8.8	11.8
J	6.1	2.0	6.0	3.0	10.5	5.0	7.2

SD=standard deviation; CI=confidence interval.

Average measured catheter displacement between day 1 and day 2 was 4.1 mm (2.7 mm for the BM method and 5.5 mm for the COGM method). The range of measured displacements was −6.0 mm to 13.5 mm for the BM method and −3.8 mm to 18.0 mm for the COGM method. For the BM method, the maximum measured catheter displacement occurred in patient C; for the COGM method, it occurred in patients C and I.

When the measurement data were converted into absolute values, average catheter displacement was 3.4 mm for the BM method and 5.6 mm for the COGM method. The catheter experiencing maximum displacement was the catheter at position 12 [Fig. 1(B)]. The catheter displacements measured using the COGM method were larger than those measured using the BM method.

**Figure 3:**
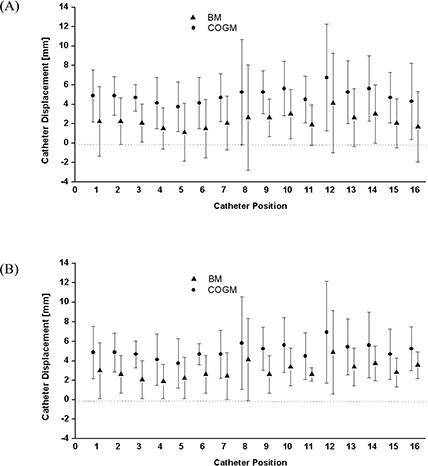
(A) Measurement of craniocaudal catheter displacement using the bony markers (BM) method and the center of two gold markers (COGM) method for 16 catheter positions. In each catheter's graph, the filled triangle and filled circle represent the mean values of the BM and COGM measurement displacements from day 1 to day 2 respectively, and the error bars show one standard deviation. (B) All data were changed into absolute values and re‐plotted.

The graph in Fig. 3(A) shows, by catheter position, the mean ± standard deviation of the catheter displacements measured using both methods in the 8 patients with 16 catheters. Fig. 3(B) re‐plots the measurement data after they were converted to absolute values. The catheters at positions 8 and 12 can be seen to be the most likely to experience the greatest displacement. These catheters are the two most anteriorly and medially located [Fig. 1(B)]. Table 2 shows the statistics for catheter displacement by catheter position.

**Table 2:** Statistics of craniocaudal catheter displacement measurement for 16 catheter positions (1 to 16)

Catheter position						95%CI	95%CI
	Mean	SD	Median	Minimum	Maximum	From	To
Measurements by the bony marker (BM) method							
1	2.3	3.6	1.5	−1.5	9.0	−0.7	5.2
2	2.3	2.4	2.3	−1.5	6.0	0.2	4.3
3	2.1	2.0	1.5	0.0	6.0	0.4	3.7
4	1.5	2.1	1.5	−1.5	4.5	−0.3	3.3
5	1.1	3.0	0.8	−3.0	6.0	−1.4	3.6
6	1.5	3.0	1.5	−4.5	6.0	−1.0	4.0
7	2.1	2.8	1.5	−1.5	6.0	−0.3	4.4
8	2.6	5.4	2.3	−4.5	13.5	−1.9	7.2
9	2.6	1.9	2.3	0.0	6.0	1.0	4.2
10	3.0	2.5	3.8	−1.5	6.0	0.9	5.1
11	1.9	2.1	3.0	−1.5	3.0	0.1	3.6
12	4.1	5.1	3.0	−3.0	15.0	−0.2	8.4
13	2.6	3.0	2.3	−3.0	6.0	0.1	5.1
14	3.0	3.0	3.8	−1.5	6.0	0.5	5.5
15	2.1	2.5	3.0	−1.5	6.0	−0.1	4.2
16	1.7	3.6	3.0	−6.0	4.5	−1.3	4.7
Measurements by the center of two gold markers (COGM) method							
1	4.9	2.7	4.5	0.8	8.3	2.6	7.1
2	4.9	2.0	5.3	1.5	7.5	3.2	6.6
3	4.7	1.4	5.3	2.3	6.0	3.5	5.8
4	4.1	2.6	4.5	0.8	8.3	1.9	6.3
5	3.8	2.5	3.8	0.0	7.5	1.6	5.9
6	4.1	2.6	4.9	−2.3	6.0	1.9	6.3
7	4.7	2.5	4.5	2.3	9.8	2.6	6.7
8	5.3	5.4	3.8	−2.3	15.8	0.7	9.8
9	5.3	2.2	4.9	2.3	9.0	3.4	7.1
10	5.6	2.8	6.0	2.3	10.5	3.3	8.0
11	4.5	2.4	5.6	0.8	7.5	2.5	6.5
12	6.8	5.5	6.4	−0.8	18.0	2.2	11.3
13	5.3	3.3	5.6	−0.8	9.0	2.5	8.0
14	5.6	3.4	6.8	0.8	9.0	2.8	8.5
15	4.7	2.6	6.0	0.8	8.3	2.5	6.9
16	4.3	3.9	5.6	−3.8	7.5	1.0	7.6

SD=standard deviation; CI=confidence interval.

In addition, the average ± standard deviation of the catheter displacement as measured by two different observers was 0.9±0.9 mm, with a maximum difference of 4.5 mm [95% confidence interval (CI): 0.8 mm to 1.1 mm], for the BM method and 1.0±0.9 mm, with a maximum difference of 5 mm (95% CI: 0.8 mm to 1.1 mm), for the COGM method.

## IV. DISCUSSION

The average displacement (4.1 mm) between the first and second fractions (on average, 19.5 hours difference) in this study is quite small as compared with the average displacement noted in several reports^(^ ^9^ ^–^ ^12^ ^)^ in the literature. Using fluoroscopy, Martinez et al.^(9)^ measured a mean displacement of 20 mm between the first and second fractions (at least 6 hours difference, and 36 hours between the first and the fourth fractions). They reported that needle movement decreased between the subsequent fractions to an average of 4 mm (between the third and fourth fractions). Using measurements of catheter tips by plain films taken before treatment, Damore et al.^(10)^ reported a mean displacement of 7.6 mm and a maximum displacement of 28.5 mm between the first and second fractions (40 hours for a total of 4 HDR fractions). They also reported a reduction in needle movement after the first day (to an average of 2 mm between the third and fourth fractions). Using a 5‐mm CT scan, Hoskin et al.^(11)^ reported that the average template movement was 1 mm and that catheter movement relative to the prostate was 9.7 mm between the first and second fractions (over a period of 18 – 24 hours). Mullokandov and Gejerman^(12)^ reported that no displacement of catheters relative to the template occurred and that the mean consecutive catheter displacement was 2, 8, and 10 mm before the second, third, and fourth fractions. Because the time interval between fractions was 6 hours in their study, the measured displacement (10 mm) before the fourth fraction (minimum 18 hours difference) can be compared with our measurement (4.1 mm) before the second fraction.

These four HDR fraction studies in the literature^(^ ^9^ ^–^ ^12^ ^)^ showed time‐dependent catheter displacement between fractions. The maximum catheter displacement occurred up to approximately 12 hours after the first fraction (20 mm before the second fraction,^(9)^ 7.6 mm before the second fraction,^(10)^ and 6 mm before the third fraction^(12)^). Displacement magnitude subsequently decreased for the following fraction.

Our two measurement methods may potentially contain some error:

1. Because of the 3‐mm CT slice thickness used, the lower limit of accuracy of our measurement is 3 mm. Even with assignment of a fractional slice value whenever the tip of a catheter was obscure on a given CT slice, the maximum error possible between the reference slice and the slice containing a catheter tip is 3 mm.
2. The gold seed markers generate artifacts. In general, a gold seed marker appears on two or three CT slices because of its size (5 mm in length, 1 mm in diameter). Two ideal scenarios are possible. First, a gold seed marker is seen as a medium‐sized bright dot on two consecutive CT slices, with the position of the seed defined as the center of these two CT slices. Second, a gold seed marker is seen over three CT slices, appearing as a big, bright dot on the middle CT slice, and as a small dot on the previous and next slices. The position of this gold seed is defined as the middle of the three CT slices. Markers usually fall between these two ideal scenarios. Hence, the maximum error from artifact of a gold seed marker is 1.5 mm.
3. In the COGM method, gold seed migration occurs. One paper in the literature^(13)^ measured gold seed migration by intermarker distance, finding that the 96th percentile value was less than 1.5 mm. In our study, the average intermarker distance variation was 1.4 mm and the 95th percentile value was 1.9 mm. We believe that migration of the center of the two gold seed markers was much less than the actual movement of the markers themselves.
4. Organ and patient movements contribute some error.
5. Error can be generated from the slanting angle of the catheters. In general, no catheter was inserted into the prostate perfectly normal to the plane of the axial CT image. The maximum angle of catheters in this study was less than 15 degree by visual measurement. This 15‐degree angle translates into a 3.5% error in the measurement of catheter depth on axial CT images.
6. Observer error.

Before considering prostate and catheter movement relative to the bony marker between fractions, several assumptions are required.

First, we found that swelling of the prostate and resolution of edema between fractions in HDR brachytherapy was insignificant^(6)^—less than 10% on average. Hence, the volume change in the prostate between fractions can be ignored, although this small change of prostate volume between fractions may cause a certain catheter displacement.

**Figure 4:**
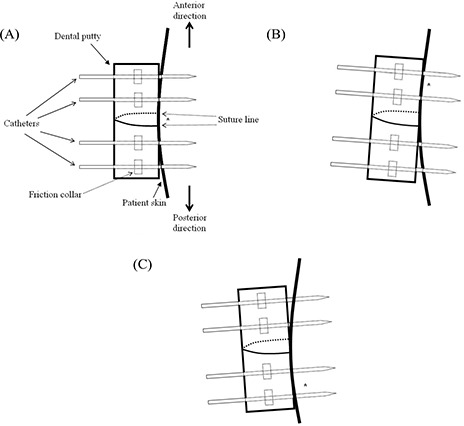
The fulcrum (denoted as *) for the rotation of putty in the lateral plane is located (A) at the centerline along the suture line on the first day. The fulcrum moves (B) in the anterior or (C) in the posterior direction of the putty between fractions.

Second, we could sometimes see individual movement of the catheters relative to the putty [Fig. 4(A)]. However, based on a physician's visual inspection before the second fraction, that event rarely happens. Consequently, catheters may be assumed to move together with the putty to explain the average catheter displacement measured by either the BM or the COGM method.

**Figure 5:**
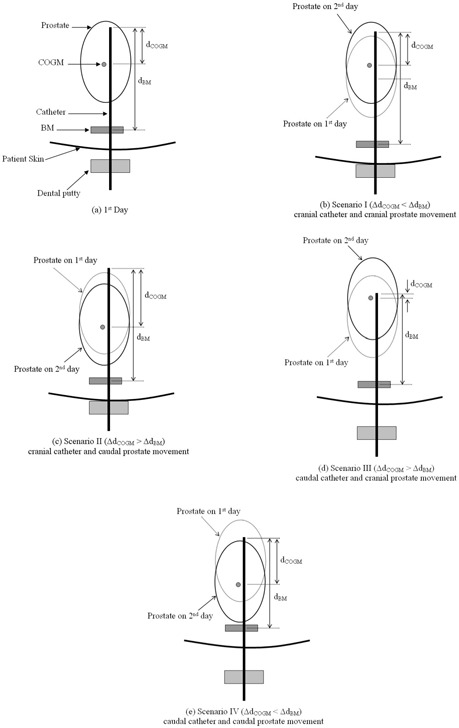
Four different scenarios for catheter and prostate movement from day 1 to day 2 relative to the bony markers (BM). Compared with day 1 [(A)], each schematic diagram on day 2 [(B), (C), (D), (E)] respectively corresponds with each scenario (I, II, III, IV), depending on the catheter and prostate movement relative to the BM. $\Delta d_{\text{BM}}$ or $\Delta d_{\text{COGM}}$ are the catheter depth differences from day 1 to day 2, measured by either BM or the center of two gold markers (COGM) method. A positive $\Delta d_{\text{BM}}$ or $\Delta d_{\text{COGM}}$ means that the catheter has moved in the inferior direction from day 1 to day 2 (caudal displacement).

Finally, movement of the OARs is being ignored, and only two movements (catheter and prostate) relative to the BM are being considered between fractions. If the prostate does not move relative to the catheters, then the catheter displacements measured by either the BM $\left(\Delta d_{\text{BM}}\right)$ or the COGM method $\left(\Delta d_{\text{COGM}}\right)$ should be the same $\left(\Delta d_{\text{BM}}=\Delta d_{\text{COGM}}\right)$. Otherwise, Fig. 5 shows the four possible alternative scenarios.

In the present study, the observed caudal displacements of the catheters resemble scenarios III [Fig. 5(D)] and IV [Fig. 5(E)]. In particular, the cases in Fig. 2 in which the average $\Delta d_{\text{BM}}$ is greater than the average $\Delta d_{\text{COGM}}$ (for patients A and G) can be classified into scenario IV. The remaining 8 cases in which the average $\Delta d_{\text{COGM}}$ is greater than the average $\Delta d_{\text{BM}}$ correspond to scenario III, depending on prostate and catheter movement relative to the bony marker. A recent study^(13)^ on prostate position relative to the pelvic bony marker also demonstrated significant interfractional movement of the prostate relative to the pelvic bony marker for external‐beam radiation therapy. Hence, we believe that the GOGM method is more accurate than the BM method in the present study.

We found that the two most anterior and medial catheters [positions 8 and 12 in Fig. 1(B)] were more likely to have a large displacement (Fig. 3). The depth of catheter positions 8 and 12 were the shallowest because the advancement of those catheters was blocked by the presence of the bladder. Although the bladder position may change depending on its fullness, these shallowest implanted catheters may be the most vulnerable to displacement between fractions. Conversely, in some cases, the posterior catheters [positions 1, 5, 9, and 13 in Fig. 1(B)] were deeply advanced into the medial inferior portion of the seminal vesicles, possibly making them less vulnerable to displacement between fractions.

**Figure 6:**
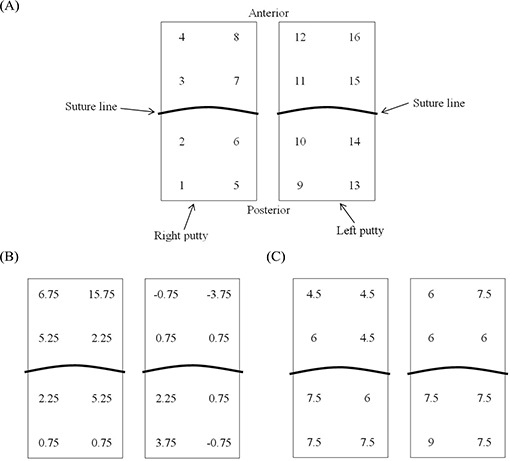
The craniocaudal catheter displacement $\left(\Delta d_{\text{COGM}}\right)$ (A) clustered into 2 groups by dental putty (schematic inferior view); (B) measured by the center of two gold markers (COGM) method for patients B; and E(C) described according to the 16 catheter positions.

Another reason for the large displacement of the shallowest implanted catheters may be rotation of the dental putty in the lateral plane (Fig. 4). The fulcrum for the rotation of the dental putty is located along the suture [Fig. 4(A)]. Between fractions, the fulcrum can move either in the anterior [Fig. 4(B)] or posterior [Fig. 4(C)] direction, depending on movement of the patient's legs. Looking at individual catheter displacement measured by COGM $\left(\Delta d_{\text{COGM}}\right)$ for patient B [Fig. 6(B)], who had the greatest variation in catheter displacement [Fig. 2], catheter positions 4 and 8 show larger catheter displacement because the right putty moved on the fulcrum in the posterior direction [Fig. 4(C)]. The fulcrum for the left putty is located at the anterior portion of the putty [Fig. 4(B)] and thus the catheter In position 9 shows the largest catheter displacement.

For patient E, who had the smallest variation in catheter displacement [Fig. 2], the fulcrum is located at the anterior portion [Fig. 4(B)] of both the right and left putties [Fig. 6(C)]. Accordingly, the catheter positions at the posterior of the putty (1, 5, 9, and 13) show larger catheter displacement.

As previously mentioned, individual catheter movement is also expected whenever the friction collar [Fig. 4(A)] of a catheter is not perfectly secured with the dental putty. This phenomenon can be observed at catheter positions 7 and 13 in Fig. 6(B) and 16 in Fig. 6(C), which deviate from the typical trend of catheter displacement because of the movement of the fulcrum. In the present study, the change of prostate volume between fractions was ignored in the catheter displacement scenarios because of insignificant magnitude. However, partial swelling or shrinking of prostate between fractions may also cause an individual catheter displacement between fractions.

We believe that large catheter movement related to catheter position can be avoided by applying more tension to the region with a change in the placement of the sutures. For instance, the suture can be placed on the putty in the superior–inferior direction instead of the current lateral direction. Another remedy is to use two lateral suture lines (one at the anterior portion of putty and the other at the posterior portion of the putty) rather than one suture line in the middle of the putty. The additional lateral suture is a promising approach to more tightly fix the putty to the perineum, where additional sutures to the four corners might not be appropriate as for a conventional prefabricated rigid plastic template.

Reports in the literature^(^ ^11^ ^,^ ^12^ ^)^ that used axial CT images for treatment planning note dose variation attributable to catheter displacement during fractions: a median 9.7‐mm catheter displacement reduced the $D_{90}$ (the dose received by 90% of the target volume) by 40%,^(11)^ and a median 9‐mm catheter displacement caused 35% of change in the dose to 90% of the prostate volume.^(12)^ In those studies, the dosimetric impact of catheter displacement was significant because the magnitude of catheter displacement was almost twice the spacing of the consecutive dwell positions (5 mm). However, in the present study, a dosimetric analysis between fractions was not feasible because of the absence of contours for target and OARs on the day 2 CT scan. The delineation of the target on CT slices has inter‐observer and intra‐observer variation and, based on reports in the external‐beam radiation therapy literature, can sometimes be overestimated by as much as 30%.^(^ ^14^ ^–^ ^18^ ^)^ As prostate movement is observed relative to catheter displacement, we can also imagine the interfractional movement of critical organs such as bladder and rectum (the urethra may move together with the prostate). The uncertainty attributable to the delineation of the target and OARs on CT images can also make a contribution to dose variation between fractions. Therefore, the dosimetric impact of the small catheter displacement (approximately 4 mm) between fractions in this study should be distinguished from the interfractional uncertainty of organ contouring on CT images. In the future, we may be able to investigate the dosimetric impact of interfractional catheter displacement and all organ movement by using MRI images to precisely contour the prostate and OARs.

In measuring catheter displacement between fractions, the 3‐mm CT slices used in the present study were comparable to the 3‐mm slices used in one study^(11)^ and the 2‐mm and 5‐mm CT scans in another study^(12)^. The average catheter displacement was quite different: approximately 4 mm in our study as compared with approximately 10 mm in others. The 3‐mm CT slices in the present study may lead to measurement error of the same range as the measured catheter displacements. However, our method in the present study of assigning fractional slice values to obscured catheter tips, artifacts of the gold seed markers, and the bony marker can reduce measurement error by half. The results of the inter‐observer variability study showed the typical range of measurement error. Although errors of more than 3 mm were observed for a few catheters, the error for most catheters was less than 1.5 mm. The average error was less than 1 mm, and the upper limit of the 95% confidence interval was 1.1 mm. Therefore, the 3‐mm CT scan in this study does not have a significant effect on measurement accuracy. The overall uncertainty of the present study is caused primarily by the CT imaging technique. Finer CT spacing and thickness (for example, 1×1 mm) could be used to reduce the systemic error of measurement.

## V. CONCLUSIONS

To summarize, we measured the craniocaudal catheter displacement in TRUS‐guided 2‐fraction HDR prostate brachytherapy, with the use of dental putty for fixation of catheters. Two measurement methods—based either on BM or on COGM—were employed. The average caudal displacement with these techniques was 4.1 mm, which is smaller than that seen with the conventional technique using a prefabricated template. The relationship between the BM and COGM measurement methods demonstrated prostate and catheter movement relative to the bony marker between fractions. Rotational movement over the fulcrum of the dental putty between fractions resulted in larger displacements for the catheters at the anterior and posterior portions of the putty—in particular, catheters 8 and 12.

## Supporting information

Supplementary Material Files

## References

[1] Martin T , Kolotas C , Dannenberg T , et al. New interstitial HDR brachytherapy technique for prostate cancer: CT based 3D planning after transrectal implantation. Radiother Oncol. 1999;52(3):257–260. 10580873 10.1016/s0167-8140(99)00113-9

[2] Lachance B , Beliveau‐Nadeau D , Lessard E , et al. Early clinical experience with anatomy‐based inverse planning dose optimization for high‐dose‐rate boost of the prostate. Int J Radiat Oncol Biol Phys. 2002;54(1):86–100. 12182978 10.1016/s0360-3016(02)02897-3

[3] Lessard E , Hsu IC , Pouliot J . Inverse planning for interstitial gynecologic template brachytherapy: truly anatomy‐based planning. Int J Radiat Oncol Biol Phys. 2002;54(4):1243–1251. 12419454 10.1016/s0360-3016(02)03802-6

[4] Lessard E , Pouliot J . Inverse planning anatomy‐based dose optimization for HDR‐brachytherapy of the prostate using fast simulated annealing algorithm and dedicated objective function. Med Phys. 2001;28(5):773–779. 11393472 10.1118/1.1368127

[5] Pouliot J , Kim Y , Lessard E , Hsu IC , Vigneron DB , Kurhanewicz J . Inverse planning for HDR prostate brachytherapy used to boost dominant intraprostatic lesions defined by magnetic resonance spectroscopy imaging. Int J Radiat Oncol Biol Phys. 2004;59(4):1196–1207. 15234056 10.1016/j.ijrobp.2004.02.055

[6] Kim Y , Hsu IC , Lessard E , Vujic J , Pouliot J . Dosimetric impact of prostate volume change between CT‐based HDR brachytherapy fractions. Int J Radiat Oncol Biol Phys. 2004;59(4):1208–1216. 15234057 10.1016/j.ijrobp.2004.02.053

[7] Kim Y , Hsu IC , Lessard E , Pouliot J , Vujic J . Dose uncertainty due to computed tomography (CT) slice thickness in CT‐based high dose rate brachytherapy of the prostate cancer. Med Phys. 2004;31(9):2543–2548. 15487736 10.1118/1.1785454

[8] Pickett B , Pouliot J . Prostate brachytherapy. In: Van Dyk J, ed. Modern technology of radiation oncology. Vol 2 Madison (WI): Medical Physics Publishing; 1999:387–421.

[9] Martinez AA , Pataki I , Edmundson G , Sebastian E , Brabbins D , Gustafson G . Phase II prospective study of the use of conformal high‐dose‐rate brachytherapy as monotherapy for the treatment of favorable stage prostate cancer: a feasibility report. Int J Radiat Oncol Biol Phys. 2001;49(1):61–69. 11163498 10.1016/s0360-3016(00)01463-2

[10] Damore SJ , Syed AM , Puthawala AA , Sharma A . Needle displacement during HDR brachytherapy in the treatment of prostate cancer. Int J Radiat Oncol Biol Phys. 2000;46(5):1205–1211. 10725633 10.1016/s0360-3016(99)00477-0

[11] Hoskin PJ , Bownes PJ , Ostler P , Walker K , Bryant L . High dose rate afterloading brachytherapy for prostate cancer: catheter and gland movement between fractions. Radiother Oncol. 2003;68(3):285–288. 13129636 10.1016/s0167-8140(03)00203-2

[12] Mullokandov E , Gejerman G . Analysis of serial CT scans to assess template and catheter movement in prostate HDR brachytherapy. Int J Radiat Oncol Biol Phys. 2004;58(4):1063–1071. 15001246 10.1016/j.ijrobp.2003.08.020

[13] Schallenkamp JM , Herman MG , Kruse JJ , Pisansky TM . Prostate position relative to pelvic bony anatomy based on intraprostatic gold markers and electronic portal imaging. Int J Radiat Oncol Biol Phys. 2005;63(3):800–811. 16199313 10.1016/j.ijrobp.2005.02.022

[14] Hoffelt SC , Marshall LM , Garzotto M , Hung A , Holland J , Beer TM . A comparison of CT scan to transrectal ultrasound–measured prostate volume in untreated prostate cancer. Int J Radiat Oncol Biol Phys. 2003;57(1):29–32. 12909212 10.1016/s0360-3016(03)00509-1

[15] Rasch C , Barillot I , Remeijer P , Touw A , van Herk M , Lebesque JV . Definition of the prostate in CT and MRI: a multi‐observer study. Int J Radiat Oncol Biol Phys. 1999;43(1):57–66. 9989514 10.1016/s0360-3016(98)00351-4

[16] Roach M 3rd , Faillace‐Akazawa P , Malfatti C . Prostate volumes and organ movement defined by serial computerized tomographic scans during three‐dimensional conformal radiotherapy. Radiat Oncol Investig. 1997;5(4):187–194. 10.1002/(SICI)1520-6823(1997)5:4<187::AID-ROI4>3.0.CO;2-U 9327498

[17] Roach M 3rd , Faillace‐Akazawa P , Malfatti C , Holland J , Hricak H . Prostate volumes defined by magnetic resonance imaging and computerized tomographic scans for three‐dimensional conformal radiotherapy. Int J Radiat Oncol Biol Phys. 1996;35(5):1011–1018. 8751410 10.1016/0360-3016(96)00232-5

[18] Wachter S , Wachter‐Gerstner N , Bock T , et al. Interobserver comparison of CT and MRI‐based prostate apex definition. Clinical relevance for conformal radiotherapy treatment planning. Strahlenther Onkol. 2002;178(5):263–268. 12082686 10.1007/s00066-002-0907-x

