# Supplementary material for: Measurement of craniocaudal catheter displacement between fractions in computed tomography–based high dose rate brachytherapy of prostate cancer
Source: J Appl Clin Med Phys. 2007 Sep 17;8(4):1–13. doi: 10.1120/jacmp.v8i4.2415 (PMC5722614; doi:10.1120/jacmp.v8i4.2415)
Supplement: Supplementary file 1 — Supplementary Material Files [file ACM2-8-001b-s001.pdf]

Measurement of cranio-caudal catheter displacement between fractions in CT-based HDR brachytherapy of prostate cancer

Abstract: The objective of this work is to measure the cranio-caudal displacement of catheters occurring between consecutive fractions of transrectal ultrasound (TRUS) guided high dose rate (HDR) prostate brachytherapy. Ten consecutive patients were treated with 2 fractions of 9.5 Gy TRUS guided HDR brachytherapy using dental putty for the fixation of catheters. For each patient, a CT scan with 3 mm slice thickness was acquired before each of the two fractions. Two different references were employed to measure the catheter displacement between fractions: the ischial bone as a bony marker (BM) and the center of two gold markers (COGM) implanted in the prostate. The catheter displacement was calculated by multiplying the thickness of CT slice with the difference in number of CT slices between the reference slice and the slice containing the tip of a catheter. The average (range) magnitude of caudal catheter displacement was 2.7 mm (-6.0 to 13.5 mm) for BM method and 5.4 mm (-3.75 to 18.0 mm) for COGM method, respectively. The measurement data obtained from BM and COGM methods verified that both prostate movement and catheter displacement occurred independently between fractions. The most anterior and medial two catheters (catheter position 8 and 12) had the greatest tendency to be displaced in the caudal direction because they were located at the most distant position from the fulcrum,

susceptible to the rotation of the dental putty in lateral plane due to the movement of patient legs between fractions. In conclusion, the use of both BM and COGM methods can demonstrate the prostate and catheter movement relative to the BM between fractions. We found a pattern of catheter displacement using our technique. Based on our finding further improvement of our results may be possible by modification of our current technique.

Key words: Catheter displacement, HDR brachytherapy, prostate cancer, bony marker, center of two gold markers

PACS number: 87.53.Jw

I. INTRODUCTION

High dose rate (HDR) brachytherapy can deliver very conformal radiation dose to prostate with catheters inserted into the tumor. Recently, computed tomography (CT) and magnetic resonance imaging (MRI) were introduced into HDR brachytherapy planning. The anatomical information is displayed along with the dose distribution within the target and organs at risk (OARs). Three dimensional treatment planning significantly improves display of dosimetric information and allows adjustment of dwell times to improve coverage of the target while sparing critical organs adjacent to the target [(1)]. Another advancement made in HDR brachytherapy is the development of the inverse planning software which allows the optimization of dwell time distribution providing the desired dose distribution based on the prescribed dose constraints [(2-4)]. Furthermore, functional imaging information from MR spectroscopy can be used for treatment planning to better identify dominant intraprostatic malignant lesions [(5)]. Despite the advantages mentioned above, dose uncertainties still remain in the HDR brachytherapy for the prostate cancer. We recently addressed the dosimetric

impact of prostate volume change due to the trauma caused by the insertion of catheters together with the resolution of edema between fractions [6] and the dose uncertainty due to the intrinsic characteristics of finite thickness of CT slice [7]. This translates into a discrepancy between the source dwell positions observable on planning CT or MRI images and the actual dwell positions during the dose delivery by the afterloader.

In this study, the cranio-caudal catheter displacement between fractions due to patient and prostate motions was measured by acquiring CT scans before each fraction for 10 patients.

II. METHODS AND MATERIALS

For this study, 10 consecutive patients (later referred to as patient A to J) were recruited. 16 catheters were implanted for each patient except for 2 patients: patient A (14 catheters) and patient I (18 catheters). Total number of catheters depended on the prostate size and the need to cover the entire tumor volume. The size of prostate was determined from the planning target volume (PTV) in CT based HDR planning procedure. The mean ± standard deviation values for the volume of prostate implanted with 16 catheters were 37.7 cc ± 6.2 cc. The prostate volume for patient A and I was 23.1 cc and 136.1 cc, respectively.

Deleted: Sixteen

A. Treatment procedures

In our institute, HDR prostate brachytherapy boost is performed in two 9.5 Gy fractions after 45 Gy of external beam radiotherapy. During the procedure, a physician inserts Flexi-guide catheters (Best Medical, Springfield, VA) into the prostate using a freehand transrectal ultrasound (TRUS) guided technique. An in-house customized catheter fixation technique using dental putty (Fig. 1(a)) [8] was developed and used instead of conventional pre-fabricated template. The freehand TRUS guided technique gives more freedom of catheter distribution adapted to the patient’s anatomy compared with the conventional pre-fabricated

template method. Hence, the catheters can be closer together at some level and further apart at other level to increase dose conformality to target while sparing normal structures. The entire treatment procedure, consisting of catheter insertion, CT based treatment planning (Plato version 14.2 Nucletron, The Netherlands), and treatment delivery, is performed over 24 hours. The first fraction is delivered in the early afternoon on the first day, while the second fraction is delivered in the morning of the second day [6, 8]. A single treatment plan is used for both fractions. The treatment plans are obtained with our in-house anatomy based inverse planning algorithm (IPSA, Inverse Planning based on Simulated Annealing), which optimizes the dwell times once the dose constraints and the prescription are specified [2-4].

B. General pattern of 16 catheters inserted into prostate

As seen in Fig. 1(b), in general 16 catheters were inserted into the prostate in four rows by four columns. The labeling was from right first to left fourth column. The numbering scheme of catheters is also shown in Fig. 1(b). The catheters fixed by the dental putty were not parallel as in the conventional pre-fabricated template technique. They sometimes converged or diverged to cover the entire target volume based on TRUS image. In Fig. 1(b) the midline of the prostate was satisfactorily covered by the catheters on the second (position 5 to 8) and third column (position 9 to 12) even though no catheters were located at the midline of perineum in Fig. 1(a).

Deleted: seen

C. Measurement of cranio-caudal catheter displacement

In order to measure the cranio-caudal catheter displacement between fractions, a second CT scan with 3 mm slice spacing and thickness (SOMATOM Emotion, Siemens Medical Solutions, Malvern, PA) was obtained for each patient with implanted catheters on the morning of the second day prior to the delivery of the second treatment. The spiral CT modality was used for the pelvic scan with 3 mm collimation and reconstruction thickness, 6

mm/rotation bed speed, one second gantry rotation period. The CT gantry angle was 0 degree, the field of view was 15 x 15 cm and the image resolution was 1024 x 1024. For the pelvic CT scan, the patient setup was head first in supine position with pillow under the knee and hands on chest.

All of the catheter displacement measurements on the first and second day axial CT scans were made by one observer. The tip of the catheters was identified by locating the end of air column in the catheter on the CT image. The air column in a catheter is displayed as a black dot on axial CT image. Sometimes, on the last axial CT image containing the tip of a catheter, the size of black dot was smaller than expected because the reconstruction volume for that CT slice did not fully contain the tip of the catheter. Hence, the tip of the catheter was assumed to be located between the current slice and the previous one [7]. For instance, if the size of black dot shown on (i)th CT slice that contains the tip of a catheter is not as big as expected, the tip of the catheter is considered to be located on the CT slice assigned half integer number such as (i)-1/2. Therefore, the catheter depth calculated on (i)th CT slice was decreased by 1.5 mm due to the half integer CT slice number assigned. The measured catheter insertion depth is the distance from the tip of each catheter to a reference CT slice. Two different references were used for each measurement. For the first method, the most inferior CT slice containing the ischial bone, bony marker (BM), was chosen as the reference CT slice. For the second method, the center of the two gold seed markers (COGM) implanted in the prostate (one is at the base and the other is at the apex of the prostate) was used to determine the reference CT slice. The displacement was calculated by multiplying the difference in catheter depths measured on the first and the second day CT scans with the 3 mm of CT slice thickness as follows.

$$(\text{Catheter Depth})_{\text{1st\_day}} = [\text{CT\_Slice}_{\text{Catheter tip}} - (\text{CT\_Slice}_{\text{GM1}} + \text{CT\_Slice}_{\text{GM2}})/2]_{\text{1st\_day}} * 3\ \text{mm}$$

$$(\text{Catheter Depth})_{\text{2nd\_day}} = [\text{CT\_Slice}_{\text{Catheter tip}} - (\text{CT\_Slice}_{\text{GM1}} + \text{CT\_Slice}_{\text{GM2}})/2]_{\text{2nd\_day}} * 3\ \text{mm}$$

$$\text{Catheter displacement} = (\text{Catheter Depth})_{\text{2nd\_day}} - (\text{Catheter Depth})_{\text{1st\_day}}$$

Deleted: air space

Deleted: due to volume averaging and then

Deleted: the

Deleted: .

In this study, a positive displacement means the catheter has moved inferiorly from day 1 to day 2 (caudal displacement), and a negative displacement means the catheter has moved deeper into the patient superiorly (cranial displacement). The measurements were analyzed using descriptive statistics for each patient and each catheter position. For the total of 160 catheters, the measurement was performed again by another observer to assess inter-observer differences in the measured catheter displacement.

III. RESULTS

The measurement data are displayed in Fig. 2 with mean ± standard deviation value and summarized in Table 1 for all 10 patients. All measurement data were changed into the absolute values and replotted as Fig. 2(b). The average catheter displacement between the first day and the second day was 4.1 mm (2.7 mm for BM and 5.5 mm for COGM measurement, respectively). The range of measured displacements was -6.0 to 13.5 mm for BM measurement and -3.8 to 18.0 mm for COGM measurement. The maximum catheter displacement was observed in patient C for the BM measurement method and it was observed in patient C and I for the COGM measurement method. For the converted measurement data in the absolute values, the average catheter displacement was 3.4 mm for BM and 5.6 mm for COGM measurement method, respectively. The catheter experiencing the maximum displacement was stationed at the twelfth catheter position (Fig. 1(b)). The measured catheter displacements were greater when they were based on COGM measurement.

Deleted: occurred

Deleted: when using

Deleted: ,

Deleted: while

Deleted: when using

In Fig. 3, catheter displacements measured with both methods for 8 patients who had 16 catheters are represented with mean ± standard deviation value corresponding to their catheter position. In addition, the measurement data were changed into absolute values and replotted as Fig. 3(b). One can observe that catheter position 8 and 12 were most likely to have the greatest displacement. These two catheters correspond to the two most anteriorly and

medially located catheters (Fig. 1(b)). Table 2 shows the statistics for the catheter displacement depending on the catheter position.

In addition, the average ± standard deviation value in the difference of catheter displacement measured by two different observers was 0.9 ± 0.9 mm with maximum of 4.5 mm (95% confidence interval: 0.8 – 1.1 mm) for BA method and 1.0 ± 0.9 mm with maximum of 5 mm (95% CI: 0.8 – 1.1 mm) for COGM method, respectively.

Deleted: 2
Deleted: 89
Deleted: 78
Deleted: 06
Deleted: 0.98
Deleted: 4
Deleted: 2

IV. DISCUSSION

The average displacement (4.1 mm) between the first and the second fraction (on average, 19.5 hours difference) in this study is quite small compared with several reports [(9-12)] in the literature. Martinez et al. [(9)] measured a mean displacement of 20 mm using fluoroscopy between the first and the second fraction (at least 6 hours difference and 36 hours between the first and the fourth fraction). They reported the needle movement decreased between the subsequent fractions to an average of 4 mm (between the third and fourth fraction). Damore et al. [(10)], using measurements of catheter tips done for plain films prior to treatment, reported a mean displacement of 7.6 mm and a maximum displacement of 28.5 mm between the first and the second fraction (40 hours for total 4 HDR fractions). They also reported a decrease in needle movement after the first day (to an average of 2 mm between the third and the fourth fraction). Hoskin et al. [(11)] reported that the average template movement was 1 mm and the catheter movement relative to the prostate was 9.7 mm, using 5 mm CT scan, between the first and second fraction (over 18 – 24 hours). Mullokandov and Gejerman [(12)] reported that there was no displacement of catheters relative to the template and the mean consecutive catheter displacement was 2, 8 and 10 mm for before the second, third and fourth fraction. Because the time interval between fractions was 6 hours in their study, the displacement before the fourth fraction (minimum 18 hours difference) in their measurement (10 mm) can

be compared with our measurement before the second fraction (4.1 mm). The four fraction HDR studies [9, 10, 12] in the literature showed a time dependent fashion of catheter displacement between fractions. The maximum catheter displacement occurs up to ~ 12 hours after the first fraction (20 mm before the second fraction [9], 7.6 mm before the second fraction [10], and 6 mm before the third fraction [12], respectively) and its magnitude is subsequently decreased for the following fraction.

Our two measurement methods may contain some potential error.

1. Because of the thickness of the CT slices used, the lower limit of accuracy of our measurement is 3 mm. Even if the half integer was assigned whenever the tip of catheter was obscure on a CT slice, the possible maximum error between a reference slice and the slice containing a catheter tip is 3 mm.
2. Artifacts generated from our gold seed markers. In general, a gold seed marker appears over 2 or 3 CT slices because its dimension was 5 mm in length and 1 mm in diameter. There are 2 possible ideal scenarios. First, a gold seed marker is seen as a medium size of bright dot on 2 consecutive CT slices in which the position of a gold seed is defined as the center of the 2 CT slices. Second, when it is seen over 3 CT slices, a gold seed marker appears as a big bright dot on middle CT slice and a small dot on the previous and next CT slices in which the position of gold seed was defined as the middle CT slice. A gold seed marker is usually between two ideal scenarios. Hence, the maximum error from artifact of a gold marker seed is 1.5 mm.
3. Gold seed migration in the COGM measurement method. In a literature [13], the gold seed migration was measured by the inter-marker distance and its 96 percentile value was less than 1.5 mm. In our study, the average inter-marker distance variation was 1.4 mm and the 95% percentile value was 1.9 mm. We believe that the migration of

the center of two gold seed markers was much less than the actual movement of two gold markers.

4. Organ and patient movements.

5. Error generated from slanting angle of the catheters. In general, a catheter was not inserted into the prostate perfectly normal to the plane of axial CT image. The maximum slanting angle of catheters in this study was less than 15 degree when it was visually measured. This 15 degree slanting angle of a catheter is translated into 3.5 % error in the measurement of the catheter depth using axial CT images.

6. Observer's error

Deleted: 3

Deleted: 4

Deleted: comparing two CT scans obtained by different CT gantry angles¶
5

Prior to considering prostate and catheter movement relative to the BM between fractions, several assumptions are required. First, we found that the swelling of prostate and resolution of edema between fractions in HDR brachytherapy [6] was insignificant, less than 10 % on average. Hence, the volume change of prostate between fractions can be ignored though this small change of prostate volume between fractions may cause a certain catheter displacement. Second, we could sometimes see the individual movement of catheters relative to the putty (Fig. 5(a)). However, this event rarely happens based on a physician's visual inspection before the second fraction. Consequently, catheters may be assumed to move together with the putty to explain the average catheter displacement measured by either BM or COGM method. Finally, in case we can ignore the movement of OARs, we may consider only two movements (catheter and prostate movement) relative to the BM between fractions. If there is no movement of prostate relative to the catheters, the catheter displacements measured by either BM or COGM method (Δd_{BM} or Δd_{COGM}) should to be the same (Δd_{BM} = Δd_{COGM}). Otherwise, four possible scenarios are shown in Fig. 4. In this study, the caudal catheter displacements observed are similar to the scenario III (Fig. 4(d)) and IV (Fig. 4(e)). In particular, in Fig. 2 the cases in which the average Δd_{BM} is greater than the average Δd_{COGM}

(for patient A and G) can be classified into the scenario IV in Fig. 4(e) while the remaining 8 cases in which the average Δd_{COGM} is greater than the average Δd_{BM} correspond to the scenario III in Fig. 4(d), depending upon the prostate and catheter movement relative to the BM. A recent study [(13)] on prostate position relative to the pelvic bony marker (BM) also demonstrated significant interfractional movement of prostate relative to the pelvic BM for external beam radiation therapy. Hence, we believe GOGM method is more accurate than BM method in this study.

We found that the two most anterior and medial catheters (position 8 and 12 in Fig. 1(b)) were more likely to have a large displacement (Fig. 3). The depth of catheter position 8 and 12 was the shallowest because the advancement of those catheters was blocked due to the presence of bladder. Hence, these shallowest implanted catheters may be the most vulnerable to displacement between fractions. Another reason for this large displacement of these catheters may be due to the rotation of dental putty in the lateral plane (Fig. 5). The fulcrum for the rotation of dental putty is located along the suture (Fig. 5(a)). Between fractions the fulcrum can move either in the anterior (Fig. 5(b)) or posterior (Fig. 5(c)) direction, depending upon the movement of patient legs. If we look at the individual catheter displacement (Δd_{COGM}) measured by COGM in Fig. 6(a) for patient B who had the greatest variation in catheters displacement in Fig. 2, the catheter position 4 and 8 showed larger catheter displacement because the fulcrum moved in the posterior direction of the right putty in Fig. 5(c). For the left putty, the fulcrum is located at the anterior portion of the putty (Fig. 5(b)) and thus catheter position 9 displays the largest catheter displacement. For patient E who has the smallest variation in catheters displacement in Fig. 2, the fulcrum is located at the anterior portion (Fig. 5(b)) of both right and left putties in Fig. 6(c). Accordingly, the catheter positions at the posterior of the putty (1, 5, 9 and 13) show larger catheter displacement. As previously mentioned, the individual catheter movement is also expected whenever the friction collar (Fig. 5(a)) of a catheter is not perfectly secured with dental putty. This

Deleted: T

phenomenon can be observed at the catheter position 7 and 13 in Fig. 6(b) and 16 in Fig. 6(c), deviated from the typical trend of catheter displacement due to the movement of fulcrum. In this study, for catheter displacement scenarios the change of prostate volume between fractions was ignored because its magnitude was insignificant. However, partial swelling or shrinking of prostate between fractions may also cause an individual catheter displacement between fractions. We believe that the large catheter movement depending on catheter position can be avoided by giving more tension to the region by changing the placement of the sutures. For instance, the suture can be done on the putty in the superior-inferior direction instead of current lateral direction. Another remedy is the use of two lateral suture lines (one at anterior portion of putty and the other at the posterior portion of putty) in place of one suture line in the middle of putty. The additional suture on dental putty is a promising approach to tightly fix putty on the perineum while additional suture to existing four corners may not be appropriate for a conventional pre-fabricated rigid plastic template method.

In the literature [11, 12], the dose variation due to catheter displacement during fractions was reported using axial CT images for treatment planning: median 9.7 mm of catheter displacement reduced D90 (dose received by 90% of the target volume) by 40% [11] and median 9 mm catheter displacement caused 35% of change of the dose to 90% of the prostate volume [12]. In those studies, the dosimetric impact due to catheter displacement was significant because the magnitude of catheter displacement is almost twice the spacing of the consecutive dwell positions (5 mm). However, in this study, the dosimetric analysis between fractions was not feasible due to the absence of contours for target and OARs on the second day CT scan. The delineation of the target on CT slice has inter-observer and intra-observer variation and can sometimes be overestimated by as much as 30% based on external beam radiation therapy literature [14-18] . As the prostate movement is observed relative to the catheter displacement, we can also imagine the movement of critical organs such as bladder

and rectum (though urethra may move together with the prostate) between fractions. The uncertainty due to delineating target and OAR on CT images can also make a contribution to the dose variation between fractions. Therefore, the dosimetric impact due to the small catheter displacement (~ 4 mm) between fractions in this study should be distinguished from the uncertainty of organ contouring on CT images between fractions. In the future, we may be able to investigate the dosimetric impact due to catheter displacement and all organ movement between fractions by employing MRI images to contour the prostate and OARs precisely.

To measure catheter displacement between fractions, 3 mm CT scan used in this study was comparable to other studies using 3 mm CT scan [11]; 2 and 5 mm CT scans [12] even though the average catheter displacement is quite different, ~ 4 mm in our study versus ~ 10 mm for others. The 3 mm CT slice thickness in this study may lead to measurement error in the same range of measured catheter displacements. However, in this study the method of assigning half integer to the CT slice containing obscured catheter tip, artifact of gold seed marker, or inter-slice located bony marker can reduce the measurement error by half. The statistics of inter-observer variability study showed the typical range of measurement error. Although more than 3 mm error was observed for a few catheters, for most catheters the error was less than 1.5 mm. The average error was less than 1 mm and the upper 95% confidence interval value is 1.1 mm. Therefore, 3 mm CT scan in this study does not have a significant impact on measurement accuracy. The overall uncertainty of this study is primarily caused by the CT imaging technique. A fine CT spacing and thickness (e. g., 1 mm by 1 mm) can be used to reduce the systemic error of measurement.

V. CONCLUSIONS

In summary, we have measured the cranial-caudal catheter displacement in two fractional TRUS guided HDR prostate brachytherapy employing the dental putty for the fixation of catheters. Two measurement methods were employed based on either BM or COGM. The average caudal displacement using dental putty was 4.1 mm which is smaller than conventional technique using pre-fabricated technique. The relationship between BM and COGM measurement demonstrated the prostate and catheter movement relative to the BM between fractions. The movement of fulcrum for the rotation of dental putty between fractions resulted in the larger catheter displacement at anterior and posterior portion of the putty, in particular, catheter position 8 and 12.

REFERENCES

1. Martin T, Kolotas C, Dannenberg T, et al. New interstitial HDR brachytherapy technique for prostate cancer: CT based 3D planning after transrectal implantation, Radiother Oncol. 1999;52:257-260.
2. Lachance B, Beliveau-Nadeau D, Lessard E, et al. Early clinical experience with anatomy-based inverse planning dose optimization for high-dose-rate boost of the prostate, Int J Radiat Oncol Biol Phys. 2002;54:86-100.
3. Lessard E, Hsu IC, Pouliot J. Inverse planning for interstitial gynecologic template brachytherapy: truly anatomy-based planning, Int J Radiat Oncol Biol Phys. 2002;54:1243-1251.
4. Lessard E, Pouliot J. Inverse planning anatomy-based dose optimization for HDR-brachytherapy of the prostate using fast simulated annealing algorithm and dedicated objective function, Med Phys. 2001;28:773-779.
5. Pouliot J, Kim Y, Lessard E, et al. Inverse planning for HDR prostate brachytherapy used to boost dominant intraprostatic lesions defined by magnetic resonance spectroscopy imaging, Int J Radiat Oncol Biol Phys. 2004;59:1196-1207.
6. Kim Y, Hsu IC, Lessard E, Vujic J, Pouliot J. Dosimetric impact of prostate volume change between CT-based HDR brachytherapy fractions, Int J Radiat Oncol Biol Phys. 2004;59:1208-1216.
7. Kim Y, Hsu IC, Lessard E, Pouliot J, Vujic J. Dose uncertainty due to computed tomography (CT) slice thickness in CT-based high dose rate brachytherapy of the prostate cancer, Med Phys. 2004;31:2543-2548.
8. Pickett B and Pouliot J. Prostate brachytherapy. In: Van Dyk J, editor. Modern Technology of Radiation Oncology, Volume 2. Madison (WI): Medical Physics Publishing; 1999: 387-421.

9. Martinez AA, Pataki I, Edmundson G, et al. Phase II prospective study of the use of conformal high-dose-rate brachytherapy as monotherapy for the treatment of favorable stage prostate cancer: A feasibility report, Int J Radiat Oncol Biol Phys. 2001;49:61-69.
10. Damore SJ, Syed AM, Puthawala AA, Sharma A. Needle displacement during HDR brachytherapy in the treatment of prostate cancer, Int J Radiat Oncol Biol Phys. 2000;46:1205-1211.
11. Hoskin PJ, Bownes PJ, Ostler P, Walker K, Bryant L. High dose rate afterloading brachytherapy for prostate cancer: catheter and gland movement between fractions, Radiother Oncol. 2003;68:285-288.
12. Mullokandov E, Gejerman G. Analysis of serial CT scans to assess template and catheter movement in prostate HDR brachytherapy, Int J Radiat Oncol Biol Phys. 2004;58:1063-1071.
13. Schallenkamp JM, Herman MG, Kruse JJ, Pisansky TM. Prostate position relative to pelvic bony anatomy based on intraprostatic gold markers and electronic portal imaging, Int J Radiat Oncol Biol Phys. 2005;63:800-811.
14. Hoffelt SC, Marshall LM, Garzotto M, et al. A comparison of CT scan to transrectal ultrasound-measured prostate volume in untreated prostate cancer, Int J Radiat Oncol Biol Phys. 2003;57:29-32.
15. Rasch C, Barillot I, Remeijer P, et al. Definition of the prostate in CT and MRI: a multi-observer study, Int J Radiat Oncol Biol Phys. 1999;43:57-66.
16. Roach M, 3rd, Faillace-Akazawa P, Malfatti C. Prostate volumes and organ movement defined by serial computerized tomographic scans during three-dimensional conformal radiotherapy, Radiat Oncol Investig. 1997;5:187-194.
17. Roach M, 3rd, Faillace-Akazawa P, Malfatti C, Holland J, Hricak H. Prostate volumes defined by magnetic resonance imaging and computerized tomographic scans for three-dimensional conformal radiotherapy, Int J Radiat Oncol Biol Phys. 1996;35:1011-1018.

18. Wachter S, Wachter-Gerstner N, Bock T, et al. Interobserver comparison of CT and MRI-based prostate apex definition. Clinical relevance for conformal radiotherapy treatment planning, Strahlenther Onkol. 2002;178:263-268.

Figure Legends

Fig. 1. (a) Fixation of 16 catheters on the perineum using dental putty (b) Typical pattern of 16 catheters inserted into the prostate (axial CT slice for mid gland of prostate)

Fig. 2. (a) Measurement of cranio-caudal catheter displacement using BM method and COGM method for 10 patients. In each error bar graph, the triangular and circular dots represent the mean values for BM and COGM measurement displacements between day 1 and day 2, respectively, and the error bars show one standard deviation value. (b) All data were changed into absolute values and replotted.

Fig. 3. (a) Measurement of cranio-caudal catheter displacement using BM method and COGM method for 16 catheters position. In each error bar graph, the triangular and circular dots represent the mean values for BM and COGM measurement displacements between day 1 and day 2, respectively, and the error bars show one standard deviation value. (b) All data were changed into absolute values and replotted.

Fig. 4. Four different scenarios for catheter and prostate movement relative to BM between day 1 and day 2. Compared with day 1 (a), each schematic diagram (b), (c), (d) and (e) on day 2 corresponds to each scenario I, II, III and IV, respectively, depending on the catheter and prostate movement relative to the BM. Δd_{BM} or Δd_{COGM} are the catheter depth differences between day 1 and 2 measured by either BM or COGM method. A positive

Δd_{BM} or Δd_{COGM} means the catheter has moved inferiorly from day 1 to day 2 (caudal displacement).

Fig. 5. The fulcrum (denoted as *****) for the rotation of putty in the lateral plane is located at the center line along the suture line (a) on the first day while the fulcrum moves in the anterior (b) or the posterior (c) direction of the putty between fractions.

Fig. 6. The cranio-caudal catheter displacement (Δd_{COGM}) measured by COGM for patient B (b) and E (c) according to the 16 catheter positions (a) clustered into 2 groups by dental putty (schematic inferior view).

Table 1. Statistics of cranio-caudal catheter displacement measurement for 10 patients (A to J).

(A) BM method

Patient	Mean	*SD	Median	Minimum	Maximum	95% **CI (From)	95% **CI (To)
A	5.1	3.0	6.0	1.5	12.0	3.4	6.9
B	0.4	4.4	-0.8	-6.0	13.5	-2.0	2.7
C	3.8	3.4	3.0	0.0	15.0	2.0	5.6
D	0.9	2.6	0.8	-1.5	6.0	-0.5	2.3
E	3.6	1.3	3.8	1.5	6.0	2.9	4.3
F	1.6	2.2	1.5	-3.0	6.0	0.4	2.8
G	4.3	2.0	3.0	1.5	9.0	3.3	5.4
H	2.0	3.4	3.0	-4.5	6.0	0.2	3.8
I	4.0	3.6	4.5	-4.5	12.0	2.2	5.8
J	1.6	2.0	1.5	-1.5	6.0	0.5	2.7

*SD: Standard Deviation, **CI: Confidence Interval

(B) COGM method

Patient	Mean	*SD	Median	Minimum	Maximum	95% **CI (From)	95% **CI (To)
A	4.4	3.0	5.3	0.8	11.3	2.6	6.1
B	2.6	4.4	1.5	-3.8	15.8	0.3	5.0
C	6.8	3.4	6.0	3.0	18.0	5.0	8.6
D	4.7	2.6	4.5	2.3	9.8	3.3	6.1
E	6.6	1.3	6.8	4.5	9.0	5.9	7.3
F	4.6	2.2	4.5	0.0	9.0	3.4	5.8
G	3.6	2.0	2.3	0.8	8.3	2.5	4.6
H	4.2	3.4	5.3	-2.3	8.3	2.4	6.0
I	10.3	3.0	10.5	6.0	18.0	8.8	11.8
J	6.1	2.0	6.0	3.0	10.5	5.0	7.2

*SD: Standard Deviation, **CI: Confidence Interval

Table 2. Statistics of cranio-caudal catheter displacement measurement for 16 catheter positions (1 to 16)

(A) BM method

Catheter Position	Mean	*SD	Median	Minimum	Maximum	95% **CI (From)	95% **CI (To)
1	2.3	3.6	1.5	-1.5	9.0	-0.7	5.2
2	2.3	2.4	2.3	-1.5	6.0	0.2	4.3
3	2.1	2.0	1.5	0.0	6.0	0.4	3.7
4	1.5	2.1	1.5	-1.5	4.5	-0.3	3.3
5	1.1	3.0	0.8	-3.0	6.0	-1.4	3.6
6	1.5	3.0	1.5	-4.5	6.0	-1.0	4.0
7	2.1	2.8	1.5	-1.5	6.0	-0.3	4.4
8	2.6	5.4	2.3	-4.5	13.5	-1.9	7.2
9	2.6	1.9	2.3	0.0	6.0	1.0	4.2
10	3.0	2.5	3.8	-1.5	6.0	0.9	5.1
11	1.9	2.1	3.0	-1.5	3.0	0.1	3.6
12	4.1	5.1	3.0	-3.0	15.0	-0.2	8.4
13	2.6	3.0	2.3	-3.0	6.0	0.1	5.1
14	3.0	3.0	3.8	-1.5	6.0	0.5	5.5
15	2.1	2.5	3.0	-1.5	6.0	-0.1	4.2
16	1.7	3.6	3.0	-6.0	4.5	-1.3	4.7

*SD: Standard Deviation, **CI: Confidence Interval

(B) COGM method

Catheter Position	Mean	*SD	Median	Minimum	Maximum	95% **CI (From)	95% **CI (To)
1	4.9	2.7	4.5	0.8	8.3	2.6	7.1
2	4.9	2.0	5.3	1.5	7.5	3.2	6.6
3	4.7	1.4	5.3	2.3	6.0	3.5	5.8
4	4.1	2.6	4.5	0.8	8.3	1.9	6.3
5	3.8	2.5	3.8	0.0	7.5	1.6	5.9
6	4.1	2.6	4.9	-2.3	6.0	1.9	6.3
7	4.7	2.5	4.5	2.3	9.8	2.6	6.7
8	5.3	5.4	3.8	-2.3	15.8	0.7	9.8
9	5.3	2.2	4.9	2.3	9.0	3.4	7.1
10	5.6	2.8	6.0	2.3	10.5	3.3	8.0
11	4.5	2.4	5.6	0.8	7.5	2.5	6.5
12	6.8	5.5	6.4	-0.8	18.0	2.2	11.3
13	5.3	3.3	5.6	-0.8	9.0	2.5	8.0
14	5.6	3.4	6.8	0.8	9.0	2.8	8.5
15	4.7	2.6	6.0	0.8	8.3	2.5	6.9
16	4.3	3.9	5.6	-3.8	7.5	1.0	7.6

*SD: Standard Deviation, **CI: Confidence Interval
